# 

**Published:** 1874-12-01

**Authors:** 


*ORIGINAL COMMUNICATIONS:*

The Examination of the Sick; The Principles of Diagnosis; Therapeutic Methods, etc. By N. S. Davis, M.D. ... 561

On an Improved Test for Detecting Sugar in the Urine. By W. S. Haines, M.D. ... 569

*TRANSLATIONS:*

Progress of Medical Science in Germany. By E. J. Doering, M.D. ... 573

*EDITORIAL DEPARTMENT:*

The EXAMINER for the Coming Year ... 575

*CORRESPONDENCE:*

Belladonna vs. Opium. By O. T. Maxson, M.D. 576

*SOCIETY REPORTS:*

Transactions of the Chicago Society of Physicians and Surgeons, November 23, 1874, ... 578

*GLEANINGS FROM OUR EXCHANGES:*

Clinical Lecture on Croup, By Dr. H. Roger, ... 579

Puerperal Insanity. By W. W. Godding, M.D., 582

Anæsthetics ... 584

Acute Rheumatism; Pericarditis; Sudden and Rapid Effusion into Pericardium; Paracentesis Pericardii; Recovery ... 587

*BOOK REVIEWS:* ... 590

